# Solvent-Free Polycaprolactone Dissolving Microneedles Generated via the Thermal Melting Method for the Sustained Release of Capsaicin

**DOI:** 10.3390/mi12020167

**Published:** 2021-02-08

**Authors:** Jaehong Eum, Youseong Kim, Daniel Junmin Um, Jiwoo Shin, Huisuk Yang, Hyungil Jung

**Affiliations:** 1 Department of Biotechnology, Yonsei University, Seoul 03722, Korea; ejh132@yonsei.ac.kr (J.E.); ustarkim@yonsei.ac.kr (Y.K.); danny0619@yonsei.ac.kr (D.J.U.); jiwooshin@yonsei.ac.kr (J.S.); 2 JUVIC Inc., Seoul 08389, Korea; hsyang@juvicbio.com

**Keywords:** transdermal drug delivery, dissolving microneedle, sustained release, extended release, polycaprolactone, capsaicin, implantable

## Abstract

(1) Background: Dissolving microneedles (DMNs), a transdermal drug delivery system, have been developed to treat various diseases in a minimally invasive, painless manner. However, the currently available DMNs are based on burst release systems due to their hydrophilic backbone polymer. Although hydrophobic biodegradable polymers have been employed on DMNs for sustained release, dissolution in an organic solvent is required for fabrication of such DMNs. (2) Method: To overcome the aforementioned limitation, novel separable polycaprolactone (PCL) DMNs (SPCL-DMNs) were developed to implant a PCL-encapsulated drug into the skin. PCL is highly hydrophobic, degrades over a long time, and has a low melting point. Under thermal melting, PCL encapsulated capsaicin and could be fabricated into a DMN without the risk of toxicity from an organic solvent. (3) Results: Optimized SPCL-DMNs, containing PCL (height 498.3 ± 5.8 µm) encapsulating 86.66 ± 1.13 µg capsaicin with a 10% (*w*/*v*) polyvinyl alcohol and 20% (*w*/*v*) polyvinylpyrrolidone mixture as a base polymer, were generated. Assessment of the drug release profile revealed that this system could sustainably release capsaicin for 15 days from PCL being implanted in porcine skin. (4) Conclusion: The implantable SPCL-DMN developed here has the potential for future development of toxicity-free, sustained release DMNs.

## 1. Introduction

Dissolving microneedles (DMNs) are micro-sized polymeric needles that encapsulate drugs and are used for direct release of drugs into the skin with minimal skin irritation following dissolution of the polymeric needles after insertion [1]. Since DMNs deliver drugs regardless of their molecular weight in a minimally invasive manner, the DMN system has been developed as an alternative method of conventional patch and injection to deliver drugs [2,3]. Although DMN technologies have been developed to deliver various drugs such as insulin, vaccines, and minoxidil, most DMN systems focus on overcoming the stratum corneum, the outermost skin barrier, using numerous biodegradable polymers such as hyaluronic acid (HA), carboxymethyl cellulose (CMC), and polyvinylpyrrolidone (PVP) [4]. These polymers are suitable for burst release drug delivery as the drugs are released during the first hour of polymer degradation [5,6,7]. However, chronic diseases such as hypertension, chronic inflammation, or alopecia areata require a sustained drug release system on DMNs to minimize the inconvenience associated with regular, long-term drug use [8].

To achieve sustained release using DMNs, several approaches have been introduced. A nanocarrier, which coats drugs using a hydrophobic polymer by an organic solvent, has been embedded into DMNs. Thus, the release rate can be controlled by the nanocarrier structure [9], which uses the main backbone hydrophilic polymers, which provide high mechanical strength, for skin insertion, and are responsible for most DMNs. Moreover, toxicity by accumulation in cell organelles and cell death by inflammation are related to the interaction between nanoparticles and cells [10]. Furthermore, an organic solvent used during nanocarrier manufacture may cause toxicity [11]. In contrast, hydrophilic polymers, which are widely used in DMNs, were chemically modified to fabricate DMNs for sustained release because these polymers presented extended degradation times and strong mechanical strength properties [12]. This approach, however, could only elongate the biodegradable rate of DMNs from about over 10 h to a week [13,14,15,16]. Therefore, the release time of DMNs with modified polymers cannot exceed several weeks. Additionally, the multiphase nature of polymers requires further studies to explore their properties, and moreover, extended laborious procedures such as an adjustment process or stability assessment are necessary [17]. These limitations could lead to potential toxic risks and huge time-consuming developments in the application of pharmaceutics.

Biodegradable polymers, which have been used for sustained release of oral formulations, have also been applied to DMNs. Polylactic-co-glycolic acid (PLGA), consisting of polylactic acid (PLA) and polyglycolide (PGA), has been used for sustained use of bovine serum albumin over 60 days [18,19]. Another biodegradable polymer for sustained release is polycaprolactone (PCL), which is used as an implanted material for pharmaceutical applications due to its simple shape [20]. Although both PLGA and PCL could be fabricated as DMNs for sustained release of drugs, an organic solvent is still required to dissolve these polymers and mix them with the drug during the fabrication of DMNs [21,22]. Even though the volatile organic solvent seems to be completely evaporated during the fabrication process, the toxicity issue remains crucial in the pharmaceutical field, and an extended proof study is necessary [23]. For this reason, it is essential to develop DMNs without organic solvents in order to protect them from the potential risks of these solvents as well as to ensure low costs. In the case of sustained release via DMNs, rapid separation of DMNs from the patch is necessary to prevent potential risks of skin irritation and patient inconvenience since long-term release of DMNs may adhere to the skin until the drug is completely released [24].

In this study, separable PCL DMNs (SPCL-DMNs) were designed as a new sustained release drug system via DMNs without an organic solvent by using melted PCL. In this novel melting method, DMNs can be fabricated without an organic solvent by using a mixture of melted PCL and capsaicin, prepared at a temperature above the melting point of PCL (59–64 °C) [25]. Here, 65 °C was considered as the optimal temperature, as a higher temperature will further degrade the polymer and a lower temperature will not melt the polymer for the procedure. To fabricate rapidly separable SPCL-DMNs, a mixture of polyvinyl alcohol (PVA) and PVP was used as the base polymer at the bottom of the SPCL-DMN. After assessing the mechanical strength of the DMN, the separation and release profiles of this novel DMN were verified using Nile red and capsaicin, respectively. SPCL-DMNs completely left PCL with encapsulated capsaicin in the skin after insertion, thereby resulting in sustained release of the drug. This new approach offers a novel DMN fabrication method for sustained transdermal lipophilic drug delivery, without the adverse effects of organic solvents.

## 2. Materials and Methods

### 2.1. Preparation of Polydimethylsiloxane (PDMS) Mold

Sylgard 184A and Sylgard 184B (Dow Corning, Midland, MI, USA), which act as PDMS prepolymer base and curing agents, were mixed in a 10:1 weight ratio. Thereafter, the mixture was poured into the master structures, which were arranged in a 5 × 5 array containing a conical microneedle with a height of 800 µm and a base diameter of 450 µm. The PDMS molds cured at 80 °C for 1 h were peeled from the master mold and used for the fabrication of the DMN array.

### 2.2. Preparation of PCL and Base Polymer Solution

SPCL-DMNs were fabricated using two casting solutions of PCL and base polymers such as PVP, HA, and a mixture of PVA and sucrose. To prepare the blank, 500 mg of each type of PCL (80, 45, and 10 kDa) (Sigma-Aldrich, St. Louis, MO, USA) was placed in three different syringes. The syringes with PCL were sealed with parafilm and positioned in a water bath (WB-6, Daihan Scientific Co., Wonju, Korea) at 65 °C for 5 min. The syringes containing the PCL with three different molecular weights were centrifuged (ARV-310, Thinky Corp., Tokyo, Japan) at 30,835× *g* for 5 min. For SPCL-DMNs, 45 kDa PCL, 20% (*w*/*w*) capsaicin (Sigma-Aldrich, St. Louis, MO, USA), and 3% (*w*/*w*) Nile red (Sigma-Aldrich, St. Louis, MO, USA) were taken and well blended by hand in syringes, and then homogenized using a sonicator (Branson Ultrasonics Corp., Danbury, CT, USA) at 65 °C for 24 h. The base polymer was prepared using a casting solution, wherein 40% (*w*/*v*) of 32 kDa HA (Sigma-Aldrich, St. Louis, MO, USA), 40% (*w*/*v*) of 360 kDa PVP (Sigma-Aldrich, St. Louis, MO, USA), 20% (*w*/*v*) of 89–98 kDa PVA with 20% (*w*/*v*) sucrose, and 10% (*w*/*v*) PVA with 20% (*w*/*v*) PVP were dissolved in distilled water using a planetary centrifugal mixer at 53,385× *g* for 30 min.

### 2.3. Fabrication of PCL-DMNs and SPCL-DMNs

PCL-DMNs comprised a DNA array made of PCL only without a base structure at the bottom of the DMN. For the fabrication of PCL-DMNs, 70 mg PCL of three different molecular weights was placed on each mold in a Petri dish. Thereafter, Petri dishes were heated in a water bath at 65 °C for 5 min and centrifuged at 30,835× *g* for 10 min. For fabrication of SPCL-DMNs, the prepared PCL solutions, which were heated in a temperature control unit at 65 °C to maintain the viscous liquid state, were dispensed as a 5 × 5 array into the microneedle-shaped cavities of the PDMS mold using a robotic dispenser (SHOT mini 100S, Musashi Engineering Inc., Tokyo, Japan) with a 20 µm diameter nozzle. This dispensing process was performed for three different dispensing durations—0.7, 1.0, and 1.3 s—at 300 kPa pressure to control the amount of PCL solution. Next, the mold containing the PCL solution was placed in a water bath at 65 °C for 5 min. Subsequently, the PDMS mold containing the PCL solution was centrifuged for 5 min at 30,835× *g*. Moreover, the rest of the cavities in the mold were filled with a base polymer solution containing 40% (*w*/*v*) HA, 40% (*w*/*v*) PVP, or 20% (*w*/*v*) of 89–98 kDa PVA with 20% (*w*/*v*) sucrose by casting and centrifuging for 5 min at 30,835× *g*. In the case of 10% (*w*/*v*) of 89–98 kDa PVA with 20% (*w*/*v*) PVP, centrifugation was performed for 6 min at 30,835× *g*.

### 2.4. Morphology and Mechanical Strength of PCL-DMNs and SPCL-DMNs

To validate the morphological properties of the SPCL-DMN structures, the length and tip diameter of DMNs were measured using a microscope (bright field, mode, M165FC, Leica, Wetzlar, Germany). The mechanical fracture force of a single PCL-DMN and SPCL-DMN was assessed using a force machine (Z0.5TN, Zwick/Roell Inc., Ulm, Germany). In brief, individual PCL-DMNs and SPCL-DMNs were separated and fixed on the station of the force machine. The sensor probe on the machine was gradually pressed onto the DMN in a downward vertical direction at 3 mm/min, and the axial force of the DMN against the probe pressure was recorded when it touched the DMN tip until the press force reached 5 N.

### 2.5. HPLC Analysis for Capsaicin

Capsaicin was quantified using reverse-phase HPLC (Waters 600S, Waters, Milford, MA, USA) performed in a C18 column (150 mm × 4.6 i.d., Cosmosil 5C18-AR-II, Nacalai Tesque Inc., Kyoto, Japan). A stock capsaicin solution was serially diluted with methanol from 0 to 50 µg/mL (Sigma-Aldrich, St. Louis, MO, USA) to prepare a calibration curve (R2 ≥ 0.99). The analytical conditions were as follows: mobile phase, methanol/distilled water (80:20), isocratic; flow rate, 1.0 mL/min; injection volume, 10 μL; and detection wavelength, 222 nm.

### 2.6. Skin Penetration of PCL-DMNs and SPCL-DMNs

To evaluate skin penetration, a PCL-DMN array was inserted into pig cadaver skin using the homemade shooting device, which contained an injector spring to increase the shooting force by the thumb force, and then simultaneously removing PCL-DMNs from the porcine skin. To visualize skin penetration of PCL-DMNs, 1% (*w*/*v*) HA with 3% (*w*/*v*) rhodamine B (Sigma-Aldrich, St. Louis, MO, USA) in distilled water was slightly coated on the tip of the PCL-DMNs. In the case of SPCL-DMNs, PCL contained 20% (*w*/*w*) capsaicin and 3% Nile red to visualize PCL in the skin after penetration and implantation, while the analysis process was similar to that in the PCL-DMN experiment. The pig cadaver skins were imaged using a microscope (fluorescence mode, M165FC, Leica, Wetzlar, Germany).

### 2.7. Permeation of SPCL-DMNs

The in vitro permeation of SPCL-DMNs was performed using Franz diffusion cells (Hanson, Chatsworth, CA, USA) at 32 °C. SPCL-DMNs containing 20% (*w*/*w*) capsaicin were applied onto 2 × 2 cm^2^ porcine skin, which was placed onto the donor compartment of the Franz cell. After application of SPCL-DMN arrays using the implantation system on the skin, tissues were mounted onto the donor compartment of the diffusion cell. The receptor chamber was filled with PBS with 20% ethanol and maintained at 32 °C using a water jacket [26]. The test was conducted using four groups of two cells. With an occluded donor compartment, aliquots (1 mL) were withdrawn from the receptor compartment and the withdrawn volume was replaced by an equal volume of fresh PBS at predetermined time points. Samples were analyzed at fixed intervals of 0, 1, 2, 3, 4, 5, 7, 9, 11, 13, and 15 days, and released capsaicin was quantified via HPLC.

## 3. Results and Discussion

### 3.1. Fabrication and Application of SPCL-DMNs

**Figure 1:**
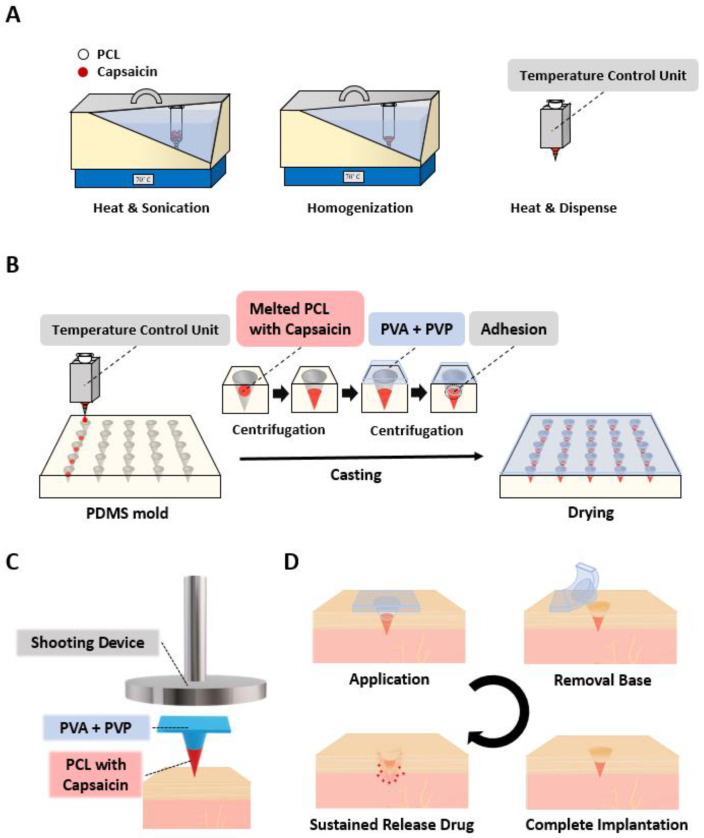
Schematic representation of fabrication of separable polycaprolactone (PCL) dissolving microneedles (SPCL-DMNs) encapsulating capsaicin. (**A**) PCL and capsaicin were drawn into a syringe and homogenized in a sonicator at 65 °C for 24 h; the temperature was controlled by a temperature control unit. (**B**) PCL and capsaicin mixtures were dispensed on the polydimethylsiloxane (PDMS) mold by a robotic dispenser at 65 °C. On the polydimethylsiloxane (PDMS) mold, melted PCL with capsaicin was placed and centrifuged to fill each microneedle-shaped cavity; next, polyvinyl alcohol (PVA) and polyvinylpyrrolidone (PVP) were cast and centrifuged to fill the remaining space in the mold with PCL. After drying, the SPCL-DMNs were peeled off from the mold. (**C**) After detachment of SPCL-DMNs from the mold, they were used directly for implantation into the skin. The SPCL-DMNs were then shot into the skin with the shooting device in the indicated alignment as illustrated in the figure. (**D**) Upon insertion, the base was manually removed for complete implantation, and the implanted PCL was degraded by interstitial fluid in the skin.

Schematic representations of the melting fabrication process of capsaicin-loaded SPCL-DMNs and their application are illustrated in Figure 1. Briefly, PCL and capsaicin were melted and homogenized in a sonicator, and the syringe was wrapped by a temperature control unit to maintain the temperature (Figure 1A). The mixture was then dispensed into the microneedle-shaped hole of the PDMS mold with arrays of 5 × 5 microneedle structures via a robotic dispenser. After the PCL tip was formed via centrifugation, the PVA with PVP mixture was cast and centrifuged to create a DMN base (Figure 1B). Physical separation of the SPCL-DMNs was achieved by introducing a hydrophilic base polymer, which had the dual function of facilitating complete insertion and separation of the PCL fraction after skin insertion. During centrifugation, the base polymer filled the remaining cavity of the mold and adhered to the PCL tip. Eventually, the array of SPCL-DMNs was detached from the mold after complete drying at room temperature for 1 day.

For application, the SPCL-DMN array was directly placed on the skin’s application site with appropriate alignment of the array, and the shooting device was positioned vertically above the SPCL-DMNs on the skin to allow targeting the bottom of the SPCL-DMNs (Figure 1C). When the SPCL-DMNs were applied to the skin using this shooting device, the base polymer could thrust the PCL fraction into the skin without fracture and separation. After inserting SPCL-DMNs into the skin, the base polymer was immediately and easily peeled off by hand because the interaction between the base polymer and PCL was low (Figure 1D). Briefly, PCL was degraded by H_2_O to 6-hydroxylcaproic acid, which then formed acetyl-CoA and further decomposed to CO_2_ and H_2_O through the citric acid cycle [27]. During this biodegradation process, an encapsulated drug was released near the application site.

### 3.2. PCL-DMNs with Different Molecular Weights

**Figure 2:**
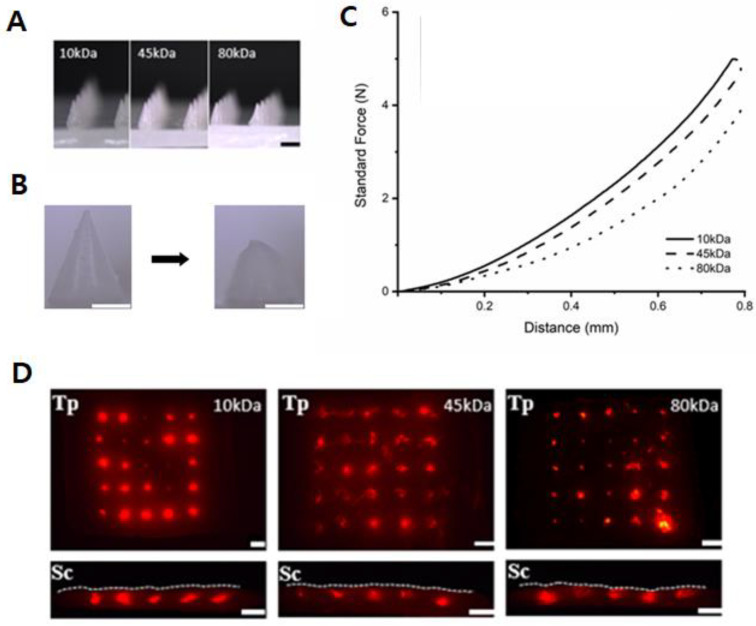
(**A**) Three different polycaprolactone dissolving microneedle (PCL-DMN) arrays generated depending on molecular weight of PCL (10, 45, and 80 kDa) (scale bar: 1 mm). The DMN array with 10, 45, and 80 kDa PCL shows a height of 772.2 ± 3.2, 772.9 ± 3.7, and 772.9 ± 4.7, respectively (*n* = 5, mean ± SEM). (**B**) Crushed dissolving microneedles after mechanical analysis (scale bar: 500 µm). (**C**) Graph illustrating mechanical strength of each array against axial force. (**D**) Top (Tp) and sectional (Sc) views of PCL-DMN array application test on porcine skin; PCL encapsulated with 1% (*w*/*w*) hyaluronic acid and 3% (*w*/*v*) rhodamine B (scale bar: 1 mm). White dashed lines represent the skin surface.

In PCL-DMNs, the pure melted PCL was used to fabric DMNs without the base polymer because verifying the moldability of the melted PCL, the shaping of DMN, and their mechanical strength are prerequisites for developing solvent-free DMNs via thermoplastic PCL. As illustrated in Figure 2A, the melted PCL could shape into DMNs regardless of the PCL molecular weight, via the melting method, thus implying that the melted PCL could be easily filled into mold cavities by dispensing and centrifugation. Moreover, this result suggests that SPCL-DMNs could also be fabricated with all molecular weights of PCL because PCL-DMN and SPCL-DMN share a similar fabrication process to shape the PCL tip, and the only difference is the existence of the base polymer in the SPCL-DMN. Furthermore, we verified the mechanical force of the PCL-DMN, which is an essential feature of the DMN to insert the skin. During force verification, PCL-DMNs were crushed (Figure 2B) while the standard force increased with the probe distance, without fracture (Figure 2C). Thus, it was inferred that PCL-DMNs represented plastic deformation; as PCL had a low elastic limit, it was permanently dented by the external force over the yield point [28]. Since the slope represented a resistance force from pressure by the steel probe, a steeper slope indicated a higher resistance force as more strength was applied to the PCL-DMNs for the same distance traveled. A higher molecular weight of PCL is presumed to present a larger mechanical force due to the molecular weight being inversely proportional to the crystallinity, where 10 kDa PCL had the highest resistance, followed by 45 and 80 kDa [29].

A fracture force of >0.058 N was applied on traditional DMNs, comprising a polysaccharide such as HA and PVP due to the crystalline structure of these polysaccharides [30,31,32,33]. PCL-DMN had no fracture; therefore, DMN arrays were applied to pig cadaver skins by the shooting device to verify the penetration of PCL-DMNs (Figure 2D). For penetration analysis, 1% (*w*/*v*) HA with 3% (*w*/*v*) rhodamine B was coated on PCL-DMNs to visualize the penetration on porcine skins. According to the top and sectional view of the porcine skin, all PCL-DMN arrays puncture the skin surface and reach the skin because rhodamine B was penetrated inside the skin through the surface, irrespective of the molecular weight.

The PCL degradation time was not clearly determined since PCL degradation was affected by various factors such as the molecular weight, shape, or environment [34]. In the case of 10 kDa, DMNs with PCL and polyethylene glycol (PEG) at a molar ratio of 1:4 revealed about 90% release in 96 h, whereas the increase in PEG simultaneously released a lower amount of the model drug [35]. This implied that the drug release time of 10 kDa PCL without PEG might be less than 96 h. In the case of 80 kDa, even up to 1-year degradation was observed [36]. In contrast, 45 kDa PCL with the hydrophobic drug imiquimod was expected to release the drug within 250 h, that is, about 10 days [37]. In this study, we encapsulated capsaicin, which could alleviate various diseases, including myofascial pain syndrome, small fiber neuropathy, rheumatoid, and inflammation, in SPCL-DMNs. Since these diseases were improved by capsaicin treatment within 7 to 14 days, 45 kDa PCL was employed to develop capsaicin-encapsulated SPCL-DMNs, even though PCL with other molecular weights can be manufactured into penetrable DMNs [38,39].

### 3.3. Optimization of Base Polymer for SPCL-DMNs

**Figure 3:**
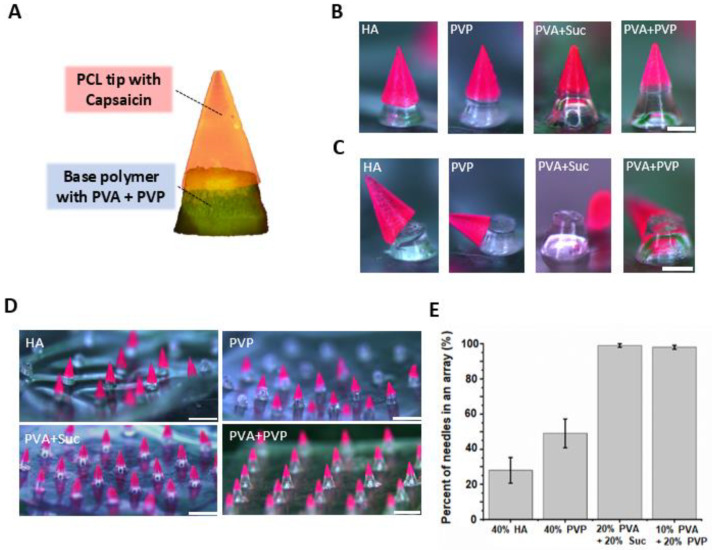
(**A**) Image of separable polycaprolactone (PCL) dissolving microneedles (SPCL-DMNs) with base polymer at the bottom; PCL contained 3% (*w*/*w*) Nile red, and the base polymer was composed of 40% (*w*/*v*) hyaluronic acid (HA) with 1 mg/mL calcein. (**B**) Fabricated SPCL-DMNs with four different base polymers, including 40% (*w*/*v*) HA, 40% (*w*/*v*) polyvinylpyrrolidone (PVP), 20% (*w*/*v*) polyvinyl alcohol (PVA) with 20% (*w*/*v*) sucrose, and 10% (*w*/*v*) PVA with 20% (*w*/*v*) PVP, and PCL containing 3% (*w*/*w*) Nile red for visible separation of PCL and base polymer (scale bar: 250 µm). (**C**) Separation images of each fabricated SPCL-DMN candidates by lateral force (scale bar: 250 µm). (**D**) SPCL-DMNs patch images with four different base polymers: 40% (*w*/*v*) HA, 40% (*w*/*v*) PVP, 20% (*w*/*v*) PVA with 20% (*w*/*v*) sucrose, and 10% (*w*/*v*) PVA with 20% (*w*/*v*) PVP after peeling-off process (scale bar: 1 mm). (**E**) Percentage of fabricated needles out of 5 × 5 arrays with four different base polymers (*n* = 4, mean ± SEM).

Since the sticky patch is usually used to deliver the drug via DMN, long-term attachment of the DMN patch on the skin is required for sustained delivery via DMN until full DMN degradation. However, attached sticky patches may cause skin irritation due to direct contact with the adhesive chemicals in the patch for hours to days. Moreover, patients can feel discomfort from the disturbance of their movement [40,41]. Therefore, rapid separation of the drug-encapsulating part of the DMN is required for the sustained delivery of DMNs to improve the convenience of patients [42]. To achieve rapid separation, we introduced a hydrophilic base layer at the bottom of the SPCL-DMN while holding the hydrophobic PCL tip at the top (Figure 3A). In this configuration, the base layer supports the insertion of the PCL tip into the dermis and separates immediately after skin insertion, owing to the low adhesion force between the PCL tip and base polymers. Therefore, the optimization of the base polymer was carefully performed considering the adhesion between PCL and the base polymer.

We employed four base polymers, which are widely used for DMNs, considering the mechanical strength and interaction between the base polymers and PCL, including 40% (*w*/*v*) HA, 40% (*w*/*v*) PVP, 20% (*w*/*v*) PVA with 20% (*w*/*v*) sucrose, and 10% (*w*/*v*) PVA with 20% (*w*/*v*) PVP. SPCL-DMNs could be fabricated with all the candidate polymers (Figure 3B). The apparently divided boundary between PCL and the base polymer was visualized with HA, PVP, and the PVA with PVP mixture. In contrast, a gradation shift at the boundary was observed in the case of PVA with sucrose. This implies that HA, PVP, and the PVA with PVP blend likely possessed higher hydrophilicity and separability than did PVA with sucrose. The PCL tip was easily separated from the base polymer when it was laterally touched by tweezers (Figure 3C). The results indicate that all PCLs were physically displaced despite the presence of the base polymers, owing to the low adhesion between the hydrophobic PCL tip and the hydrophilic base layer. 

Although all the base polymers used were suitable for fabricating DMNs and were physically separated from the PCL tip, further optimization of the base polymer selection is required for the fabrication of SPCL-DMN arrays using the molding technique. This is because base layers, which have a low adhesion force on the PCL layer, may accidentally detach during the peeling from the PDMS mold. As illustrated in Figure 3D, when HA and PVP were used as the base polymer, partial PCL tips were detached from the base polymers during the peel-off process, whereas PVA with sucrose and that with PVP could be used to fabricate 5 × 5 arrays (Figure 3D). The percentage of individual microneedles that could be fabricated into a two-layer array was calculated to determine the reproducibility of SPCL-DMNs (Figure 3E). According to the results, HA and PVP are not suitable for use as base polymers because they generate solid polymers that cause unintended detachment during the peel-off process. In contrast, mixtures of PVA combined with sucrose or with PVP produced a slightly flexible base polymer that could withstand the detachment step and could separate after the insertion step. Thus, PVA combined with sucrose or PVP was employed as a base polymer to develop SPCL-DMNs.

### 3.4. Fabrication of SPCL-DMNs with PVA and Sucrose Mixture

**Figure 4:**
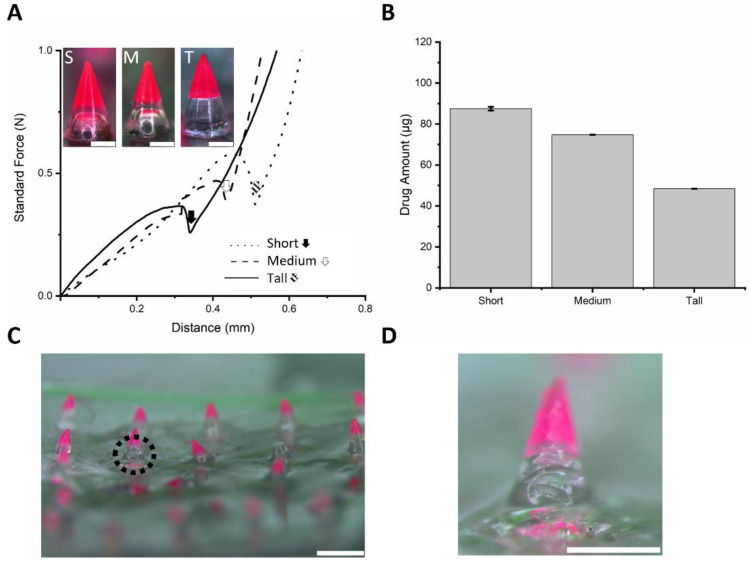
(**A**) Dissolving microneedles (DMNs) fabricated with three different ratios of polycaprolactone (PCL) and polyvinyl alcohol (PVA) with sucrose (scale bar: 250 µm). S, M, and T in the upper left indicate short, medium, and tall, respectively. Each SPCL-DMN was designed with 256.5 ± 6.0, 344.8 ± 5.4, and 377.1 ± 5.6 µm of base height, respectively (*n* = 10, mean ± SEM). The graph illustrates the standard force against press by time. Standard force was recorded by moving the probe across the axial distance; the peaks indicate the fracture force (arrow marks). (**B**) Amount of capsaicin in PCL based on length of PCL (*n* = 3, mean ± SEM). For determining the PCL quantity, 20% (*w*/*w*) capsaicin was dissolved in PCL at 65°C. The short, medium, and tall SPCL-DMNs contained 87.49 ± 0.92, 74.77 ± 0.06, and 48.43 ± 0.03 µg of PCL, respectively (*n* = 3, mean ± SEM). (**C**) Image of SPCL-DMNs with PVA and sucrose mixture after insertion using the shooting device; PCL was encapsulated with 20% (*w*/*w*) capsaicin and 3% Nile red (scale bar: 1 mm). (**D**) Magnified image of black dotted circle in Figure 4C (scale bar: 500 µm).

Since the depth of the stratum corneum is generally considered to be between 12 and 55 µm, and incomplete insertion may occur at the bottom of conventional DMNs, a sufficient base polymer length is required for complete insertion [43]. In addition, an insertion study of conventional DMNs revealed that about 300 µm of base polymer did not dissolve after 5 min of application [44]. For the development of SPCL-DMNs, a sufficient base polymer height is required to support complete PCL insertion into the skin; otherwise, PCL is exposed outside the skin. Therefore, the adequate height of the base polymer was studied with three different SPCL-DMNs (Figure 4A). For complete implantation through the stratum corneum, short, medium, and tall base heights of 256.5 ± 6.0, 344.8 ± 5.4, and 377.1 ± 5.6 µm, respectively (*n* = 10, mean ± SEM), were studied by considering 300 µm of the base polymer, which secures complete insertion of PCL. These SPCL-DMNs encapsulated 87.49 ± 0.92, 74.77 ± 0.06, and 48.43 ± 0.03 µg of capsaicin, respectively, in an array since it contained a different amount of PCL (Figure 4B). 

To verify skin penetration of SPCL-DMNs, a mechanical force experiment was performed to observe the effect of adding the base polymer to the DMN with three SPCL-DMNs comprising different heights of the base layer (Figure 4C). In this result, fractures were observed with the displacement of PCL, and thereafter, short, medium, and tall SPCL-DMNs revealed fractures of 0.381, 0.471, and 0.576 N at 0.314, 0.401, and 0.444 µm, respectively (Figure 4A, black arrow mark, white arrow mark, and slashed arrow mark). Based on the results, all conditions of the SPCL-DMNs were supposed to be skin-penetrable because fractures occurred over 0.058 N, which is the minimal requirement of the penetration force. The shorter base layer could deliver a larger amount of drug into the skin; nevertheless, there was a higher chance of incomplete separation due to the stronger adhesive force from the larger interaction between PCL and the base polymer. Since SPCL-DMNs with a PVA and sucrose mixture were considered capable of complete insertion and separation by physical analysis, they could be applied to porcine skin in order to verify their separable abilities (Figure 4C). As presumed from physical analysis, SPCL-DMNs were completely inserted in the skin; however, PCL could not be separated from the base polymer after insertion. In the black dotted circle in Figure 4C, although the base polymer was responsible for the insertion and separation of PCL, the base polymer’s morphology was modified after insertion without separation, which could lead to incomplete separation. In the enlarged image of SPCL-DMNs after insertion, wrinkling was observed on the base polymer because it absorbed water in the skin during insertion (Figure 4D). As PVA and sucrose mixtures have high water solubility, the base polymer might dissolve and increase the adhesion with PCL and thereby increase the flexibility of the polymer [45]. For this reason, the PVA and sucrose mixture was not suitable as a base polymer for SPCL-DMNs. 

### 3.5. Fabrication of SPCL-DMNs with PVA and PVP Mixture

**Figure 5:**
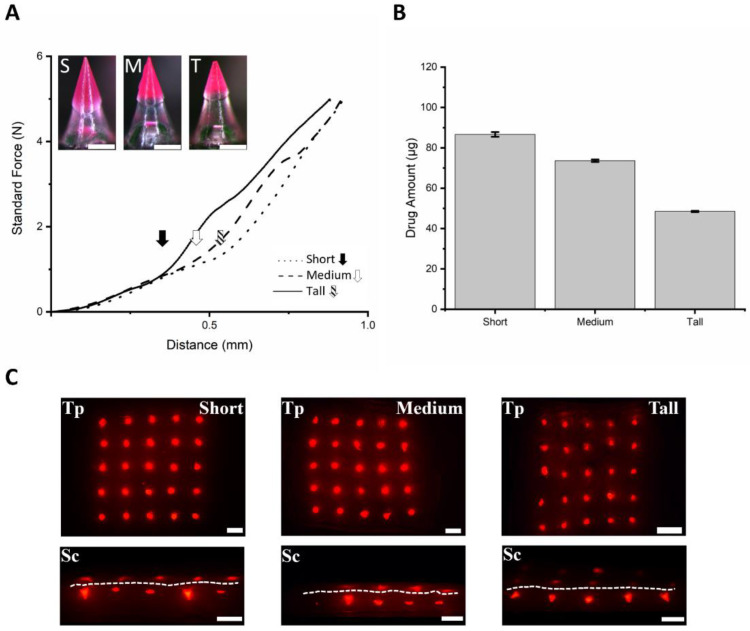
(**A**) Images of dissolving microneedles (DMNs) with three different ratios of polycaprolactone (PCL) and polyvinyl alcohol (PVA) with polyvinylpyrrolidone (PVP) (scale bar: 250 µm). S, M, and T in the upper left indicate short, medium, and tall, respectively. Each SPCL-DMN was designed with 274.6 ± 5.8, 349.1 ± 5.0, and 412.3 ± 3.8 µm of base height, respectively (*n* = 10, mean ± SEM). The graph illustrates the standard force against press by time. Standard force was recorded by moving the probe across the axial distance; the arrow marks indicate the shift of the slope. (**B**) Amount of capsaicin in PCL based on the length of PCL (*n* = 3, mean ± SEM). For determining PCL quantity, 20% (*w*/*w*) capsaicin was dissolved in PCL at 65 °C. The short, medium, and tall PCLs contained 86.66 ± 1.13, 73.62 ± 0.61, and 48.49 ± 0.30 µg of PCL, respectively (*n* = 3, mean ± SEM). (**C**) Application test of separable PCL-DMN (SPCL-DMN) array on porcine skin; the PCL encapsulated with 20% (*w*/*w*) capsaicin and 3% (*w*/*v*) Nile red. Upper left letters indicate top (Tp) and sectional (Sc) views of porcine skin. Top view images reveal that all PCL tips were placed on the skin, and sectional view shows porcine skin after application (scale bar: 1 mm). White dash lines indicate the skin surface.

The PVA and PVP mixture was a candidate base polymer that could fabricate SPCL-DMNs, and thus the mechanical strength and insertion were verified. Due to the cross-link between PVA and PVP, this blend polymer was resistant to solubility from water, hindering the dissolution of the base polymer [46]. All conditions were same as those used for the PVA and sucrose blend during fabrication. Moreover, in this experiment, three different SPCL-DMNs were designed to represent short, medium, and tall in order to determine the height of the base polymer capable of complete insertion in the skin (Figure 5A). Unlike the PVA and sucrose mixture, the clear boundary between PCL and the base polymer can potentially decrease interactions with the chemicals. A mechanical strength experiment was performed for SPCL-DMNs with PVA and PVP mixtures due to base polymer replacement (Figure 5A inset). According to the result, the standard forces of the PVA and PVP mixture increased without fracture regardless of the base height; instead, dramatic slope shifts were observed at the arrow marked points, as the machine probe reached the base polymer area. It could be inferred that the capsaicin-encapsulated part was crushed as the probe moved. The distance at the points for the slope shift appeared depending on the length of PCL. Capsaicin encapsulated in SPCL-DMNs was also examined; these SPCL-DMNs encapsulated 86.66 ± 1.13, 73.62 ± 0.61, and 48.49 ± 0.30 µg of capsaicin, respectively, in an array since PCL differed (Figure 5B).

Although all SPCL-DMNs arrays can penetrate skin because the tendency of the force analysis was similar to that of PCL-DMNs that successfully punctured pig cadaver skin, application on porcine skin was performed in order to verify the penetration and separation ability of 5 × 5 SPCL-DMN arrays encapsulating 20% (*w*/*w*) capsaicin and 3% (*w*/*w*) Nile red in PCL (Figure 5C). According to the top view of the porcine skin after insertion, all SPCL-DMN arrays implanted PCL in the skin regardless of the base height. Furthermore, the pig cadaver skin was sectionalized to ensure that the PCL parts were appropriately placed in the skin. Although partial PCL was improperly chopped to identify its whole shape, the red light emission indicated that all PCL was implanted as only PCL with Nile red could emit light. In accordance with the sectional view, contained drugs did not appear on the skin surface, which supports the precise delivery of the contained drug contrary to conventional DMNs where drugs are exposed outside of the skin during dissolution after insertion. With further research on the microneedle shape design and an additional mechanism for drug delivery, such as a micropump, SPCL-DMNs can become more precise in the drug delivery area [47,48]. 

**Figure 6:**
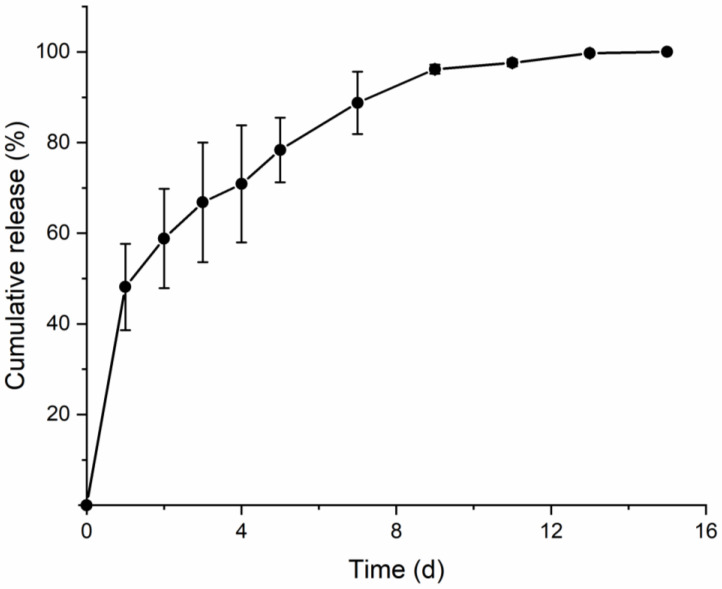
Cumulative release profile of separable polycaprolactone (PCL) dissolving microneedle (SPCL-DMN) array, with PCL encapsulating 20% (*w*/*w*) capsaicin, in porcine skin over 15 days (*n* = 2) determined using a Franz diffusion cell. Notably, 99.7% ± 0.31% of capsaicin was released within 13 days.

In the mechanical strength analysis, the PVA and PVP mixtures showed adequate insertion and separation abilities, regardless of the PCL and base polymer ratio. The developed SPCL-DMNs were expected to release the encapsulated drug following separation from the PCL after insertion into the skin. To verify and evaluate the sustained release ability of SPCL-DMNs, a Franz diffusion cell was used to insert the SPCL-DMNs into porcine skin at 32 °C. The cumulative release graph (Figure 6) revealed that 99.7% ± 0.31% of capsaicin was released within 13 days. In detail, 48.15% ± 9.51% of capsaicin permeated the skin within 1 day, about 51.54% ± 9.2% of capsaicin was released over the next 12 days, and the capsaicin was completely released in 15 days. The cumulative release pattern followed a typical sustained release profile—initial rapid release followed by slow release until complete degradation [49,50]. 

## 4. Conclusions

This study introduced novel implantable DMNs to deliver a sustained drug with long-term degradable PCL without an organic solvent. The thermal melting method was used to encapsulate capsaicin in PCL, and the resulting capsaicin-encapsulated PCL was fabricated as DMNs without the risk of potential toxicity from an organic solvent. Moreover, to facilitate direct delivery of drug-encapsulating PCL into the skin, the SPCL-DMNs were developed into physically implantable DMNs by introducing hydrophilic base polymers on the base of SPCL-DMNs. Four different polymers containing HA, PVP, PVA with sucrose mixture, and PVA with PVP mixture were analyzed for fabrication of SPCL-DMNs, where all four could produce 5 × 5 SPCL-DMN arrays. Since the PVA with PVP mixture indicated appropriate insertion and separation ability on pig cadaver skin, optimized SPCL-DMNs were developed with 10% (*w*/*v*) PVA along with a 20% (*w*/*v*) PVP mixture as a base polymer with 498.3 ± 5.8 µm of PCL encapsulating 86.66 ± 1.13 µg capsaicin. The results reveal that SPCL-DMNs exhibited sufficient mechanical strength to penetrate the skin and separate PCL in the skin. In the release profile experiment, all SPCL-DMNs contained capsaicin within 15 days. Thus, SPCL-DMNs could be used as drug delivery systems to treat chronic diseases such as hypertension and chronic inflammation, to improve “patient friendliness” and avoid irritation from chemical adhesives. This implantable SPCL-DMN fabricated using the melting method has the potential for future development into toxicity-free, sustained release DMNs.

## Data Availability

The data presented in this study are available on request from the corresponding author.

## References

[1] Lee S., Fakhraei Lahiji S., Jang J., Jang M., Jung H. (2019). Micro-Pillar Integrated Dissolving Microneedles for Enhanced Transdermal Drug Delivery. Pharmaceutics.

[2] Zhu Z., Luo H., Lu W., Luan H., Wu Y., Luo J., Wang Y., Pi J., Lim C.Y., Wang H. (2014). Rapidly dissolvable microneedle patches for transdermal delivery of exenatide. Pharm. Res..

[3] Zhao Z., Chen Y., Shi Y. (2020). Microneedles: A potential strategy in transdermal delivery and application in the management of psoriasis. RSC Adv..

[4] Wang M., Hu L., Xu C. (2017). Recent advances in the design of polymeric microneedles for transdermal drug delivery and biosensing. Lab Chip.

[5] Liu S., Wu D., Quan Y.S., Kamiyama F., Kusamori K., Katsumi H., Sakane T., Yamamoto A. (2016). Improvement of transdermal delivery of exendin-4 using novel tip-loaded microneedle arrays fabricated from hyaluronic acid. Mol. Pharm..

[6] Lee J.W., Park J.H., Prausnitz M.R. (2008). Dissolving microneedles for transdermal drug delivery. Biomaterials.

[7] Sullivan S.P., Murthy N., Prausnitz M.R. (2008). Minimally invasive protein delivery with rapidly dissolving polymer microneedles. Adv. Mater..

[8] Hong X., Wei L., Wu F., Wu Z., Chen L., Liu Z., Yuan W. (2013). Dissolving and biodegradable microneedle technologies for transdermal sustained delivery of drug and vaccine. Drug Des. Devel. Ther..

[9] Permana A.D., Tekko I.A., McCrudden M.T.C., Anjani Q.K., Ramadon D., McCarthy H.O., Donnelly R.F. (2019). Solid lipid nanoparticle-based dissolving microneedles: A promising intradermal lymph targeting drug delivery system with potential for enhanced treatment of lymphatic filariasis. J. Control. Release.

[10] Stefano D.D., Carnuccio R., Maiuri M.C. (2012). Nanomaterials Toxicity and Cell Death Modalities. J. Drug Deliv..

[11] Cadete A., Olivera A., Besev M., Dhal P.K., Gonçalves L., Almeida A.J., Bastiat G., Benoit J.P., de la Fuente M., Garcia-Fuentes M. (2019). Self-assembled hyaluronan nanocapsules for the intracellular delivery of anticancer drugs. Sci. Rep..

[12] Vora L.K., Courtenay A.J., Tekko I.A., Larrañeta E., Donnelly R.F. (2020). Pullulan-based dissolving microneedle arrays for enhanced transdermal delivery of small and large biomolecules. Int. J. Biol. Macromol..

[13] Chen B.Z., Ashfaq M., Zhu D.D., Zhang X.P., Guo X.D. (2018). Controlled delivery of insulin using rapidly separating microneedles fabricated from genipin-crosslinked gelatin. Macromol. Rapid Commun..

[14] Wang J., Ye Y., Yu J., Kahkoska A.R., Zhang X., Wang C., Sun W., Corder R.D., Chen Z., Khan S.A. (2018). Core-shell microneedle gel for self-regulated insulin delivery. ACS Nano.

[15] Wang C., Ye Y., Hochu G.M., Sadeghifar H., Gu Z. (2016). Enhanced cancer immunotherapy by microneedle patch-assisted delivery of anti-pd1 antibody. Nano Lett..

[16] Lee J., Park S.H., Seo I.H., Lee K.J., Ryu W. (2015). Rapid and repeatable fabrication of high A/R silk fibroin microneedles using thermally-drawn micromolds. Eur. J. Pharm. Biopharm..

[17] Pillay V., Seedat A., Choonara Y.E., du Toit L.C., Kumar P., Ndesendo V.M. (2013). A review of polymeric refabrication techniques to modify polymer properties for biomedical and drug delivery applications. AAPS Pharm. Sci. Tech..

[18] Makadia H.K., Siegel S.J. (2011). Poly lactic-co-glycolic acid (PLGA) as biodegradable controlled drug delivery carrier. Polymers.

[19] Park S.C., Kim M.J., Baek S.K., Park J.H., Choi S.O. (2019). Spray-formed layered polymer microneedles for controlled biphasic drug delivery. Polymers.

[20] Yu W., Jiang G., Zhang Y., Liu D., Xu B., Zhou J. (2017). Near-infrared light triggered and separable microneedles for transdermal delivery of metformin in diabetic rats. J. Mater. Chem. B.

[21] Park J.H., Allen M.G., Prausnitz M.R. (2006). Polymer microneedles for controlled-release drug delivery. Pharm. Res..

[22] Zhang Y., Chai D., Gao M., Xu B., Jiang G. (2019). Thermal ablation of separable microneedles for transdermal delivery of metformin on diabetic rats. Int. J. Polym. Mater. Polym..

[23] Paul D., Dey T.K., Dhar P., Ficai D., Grumezescu A.M. (2017). Nanoformulation and administration of PUFA-rich systems for applications in modern healthcare. Nanostructures for Novel Therapy.

[24] Bae W.G., Ko H., So J.Y., Yi H., Lee C.H., Lee D.H., Ahn Y., Lee S.H., Lee K., Jun J. (2019). Snake fang-inspired stamping patch for transdermal delivery of liquid formulations. Sci. Transl. Med..

[25] Piyasin P., Yensano R., Pinitsoontorn S. (2019). Size-controllable melt-electrospun polycaprolactone (PCL) fibers with a sodium chloride additive. Polymers.

[26] Dangol M., Yang H., Li C.G., Lahiji S.F., Kim S., Ma Y., Jung H. (2016). Innovative polymeric system (IPS) for solvent-free lipophilic drug transdermal delivery via dissolving microneedles. J. Control. Release.

[27] Heimowska A., Morawska M., Janiszewska A.B. (2017). Biodegradation of poly(ε-caprolactone) in natural water environments. Pol. J. Chem. Technol..

[28] Kurniawan D., Nor F.M., Lee H.Y., Lim J.Y. (2011). Elastic properties of polycaprolactone at small strains are significantly affected by strain rate and temperature. Proc. Inst. Mech. Eng. H.

[29] Jenkins M.J., Harrison K.L. (2006). The effect of molecular weight on the crystallization kinetics of polycaprolactone. Polym. Adv. Technol..

[30] Chen Z.H., Ren X.L., Zhou H.H., Li X.D. (2012). The role of hyaluronic acid in biomineralization. Front. Mater. Sci..

[31] Wang B., Wang D., Zhao S., Huang X., Zhang J., Lv Y., Liu X., Lv G., Ma X. (2017). Evaluate the ability of PVP to inhibit crystallization of amorphous solid dispersions by density functional theory and experimental verify. Eur. J. Pharm. Sci..

[32] Davis S.P., Landis B.J., Adams Z.H., Allen M.G., Prausnitz M.R. (2004). Insertion of microneedles into skin: Measurement and prediction of insertion force and needle fracture force. J. Biomech..

[33] Kim J.D., Kim M., Yang H., Lee K., Jung H. (2013). Droplet-born air blowing: Novel dissolving microneedle fabrication. J. Control. Release.

[34] Djonlagic J., Nikolic M.S., Sharma S.K., Mudhoo A. (2011). Biodegradable polyesters: Synthesis and physical properties. A Handbook of Applied Biopolymer Technology Synthesis, Degradation & Application.

[35] Ko P.-T., Lee I.-C., Chen M.-C., Tsai S.W. (2015). Polymer microneedles fabricated from PCL and PCL/PEG blends for transdermal delivery of hydrophilic compounds. J. Taiwan Inst. Chem. Eng..

[36] Lin W.-C., Yeh I.-T., Niyama E., Huang W.-R., Ebara M., Wu C.-S. (2018). Electrospun poly(ε-caprolactone) nanofibrous mesh for imiquimod delivery in melanoma therapy. Polymers.

[37] Romero V., Lara J.R., Otero-Espinar F., Salgado M.H., Modolo N.S.P., Barros G.A.M. (2019). Capsaicin topical cream (8%) for the treatment of myofascial pain syndrome. Rev. Bras. Anestesiol..

[38] Illigens B.M., Gibbons C.H. (2013). A human model of small fiber neuropathy to study wound healing. PLoS ONE.

[39] Deal C.L., Schnitzer T.J., Lipstein E., Seibold J.R., Stevens R.M., Levy M.D., Albert D., Renold F. (1991). Treatment of arthritis with topical capsaicin: A double-blind trial. Clin. Ther..

[40] Tokumura F., Umekage K., Sado M., Otsuka S., Suda S., Taniguchi M., Yamori A., Nakamura A., Kawai J., Oka K. (2005). Skin irritation due to repetitive application of adhesive tape: The influence of adhesive strength and seasonal variability. Skin Res. Technol..

[41] Lachapelle J.-M., Ring J., Darsow U., Maibach H., Rustemeyer T., Lachapelle J.-M., Maibach H.I. (2009). Patch testing methodology. Patch Testing and Pricking Testing: A Practical Guide Official Publication of the ICDRG.

[42] Li W., Terry R.N., Tang J., Feng M.R., Schwendeman S.P., Prausnitz M.R. (2019). Rapidly separable microneedle patch for the sustained release of a contraceptive. Nat. Biomed. Eng..

[43] Scott D.W., Miller W.H., Scott D.W., Miller W.H. (2003). Structure and function of the skin. Equine Dermatology.

[44] Demuth P.C., Garcia-Beltran W.F., Ai-Ling M.L., Hammond P.T., Irvine D.J. (2013). Composite dissolving microneedles for coordinated control of antigen and adjuvant delivery kinetics in transcutaneous vaccination. Adv. Funct. Mater..

[45] Xi H., Chen D., Lv L., Zhong P., Lin Z., Chang J., Wang H., Wang B., Ma X., Zhang C. (2017). High performance transient organic solar cells on biodegradable polyvinyl alcohol composite substrate. RSC Adv..

[46] Bernal A., Kuritka I., Saha P. (2013). Poly(vinyl alcohol)-poly(vinyl pyrrolidone) blends: Preparation and characterization for a prospective medical application. J. Appl. Polym. Sci..

[47] Vinayakumar K.B., Nadiger G., R Shetty V., Dinesh N.S., Nayak M.M., Rajanna K. (2017). Packaged Peristaltic Micropump for Controlled Drug Delivery Application. Rev. Sci. Instrum..

[48] Economidou S.N., Pissinato Pere C.P., Okereke M., Douroumis D. (2021). Optimisation of Design and Manufacturing Parameters of 3D Printed Solid Microneedles for Improved Strength, Sharpness, and Drug Delivery. Micromachines.

[49] McCormick C., Wall J.G., Podbielska H., Wawrzyńska M. (2018). Polymer-free drug-eluting stents. Functionalised Cardiovascular Stents.

[50] Szurkowska K., Laskus A., Kolmas J., Thirumalai J. (2017). Hydroxyapatite-based materials for potential use in bone tissue infections. Hydroxyapatite-Advances in Composite Nanomaterials, Biomedical Applications and its Technological Facets.

